# Strategies for High-Efficiency Mutation Using the CRISPR/Cas System

**DOI:** 10.3389/fcell.2021.803252

**Published:** 2022-02-07

**Authors:** Shuying Feng, Zilong Wang, Aifang Li, Xin Xie, Junjie Liu, Shuxuan Li, Yalan Li, Baiyan Wang, Lina Hu, Lianhe Yang, Tao Guo

**Affiliations:** ^1^ Medical College, Henan University of Chinese Medicine, Zhengzhou, China; ^2^ Department of Pharmacy, Henan University of Chinese Medicine, Zhengzhou, China

**Keywords:** CRISPR/Cas system, optimized strategies, highly efficient, mutant, off-target effect

## Abstract

Clustered regularly interspaced short palindromic repeats (CRISPR)-associated systems have revolutionized traditional gene-editing tools and are a significant tool for ameliorating gene defects. Characterized by high target specificity, extraordinary efficiency, and cost-effectiveness, CRISPR/Cas systems have displayed tremendous potential for genetic manipulation in almost any organism and cell type. Despite their numerous advantages, however, CRISPR/Cas systems have some inherent limitations, such as off-target effects, unsatisfactory efficiency of delivery, and unwanted adverse effects, thereby resulting in a desire to explore approaches to address these issues. Strategies for improving the efficiency of CRISPR/Cas-induced mutations, such as reducing off-target effects, improving the design and modification of sgRNA, optimizing the editing time and the temperature, choice of delivery system, and enrichment of sgRNA, are comprehensively described in this review. Additionally, several newly emerging approaches, including the use of Cas variants, anti-CRISPR proteins, and mutant enrichment, are discussed in detail. Furthermore, the authors provide a deep analysis of the current challenges in the utilization of CRISPR/Cas systems and the future applications of CRISPR/Cas systems in various scenarios. This review not only serves as a reference for improving the maturity of CRISPR/Cas systems but also supplies practical guidance for expanding the applicability of this technology.

## 1 Introduction

In aquatic systems, host–pathogen interactions are meaningful in the ecology and evolution of all organisms. These interactions are often characterized by a strong immune defense between prokaryotic cells (archaea) and viruses, leading to their co-evolution (England and Whitaker, 2013). The strong immune defense mechanism utilized by these organisms is known as the clustered regulatory interspaced short palindromic repeats (CRISPR) system, which is used in prokaryotes to combat a viral infection. Earlier reports of CRISPR/Cas systems report three different types: I, II, and III (Makarova et al., 2011). Each type of system is characterized by a signature protein(s). The most common type, type II CRISPR/Cas9 system, mediates the immune response in three stages as follows: (1) adaption, (2) expression, and (3) interference (Makarova et al., 2020). In the adaption stage, DNA fragments of invading plasmids or phages (termed protospacers) are incorporated into the host CRISPR locus as spacers in the form of CRISPR RNA (crRNA) repeats. In the expression stage, the precursor CRISPR RNA (pre-crRNA) molecules are processed by expressed Cas proteins and cofactors into short, mature crRNA. Next, in the interference stage, the Cas9 protein recognizes and targets the crRNA, silencing the foreign sequences (Gasiunas et al., 2012; Janik et al., 2020). Single-guide RNA (sgRNA) synthesized by crRNA and tracrRNA then guides the Cas protein to generate double-strand breaks (DSBs) three base pairs upstream from the protospacer adjacent motifs (PAM) (Jinek et al., 2012). Through this mechanism, CRISPR/Cas systems can also serve as a precise gene-editing tool for genetic manipulation.

So far, the CRISPR/Cas systems have been divided into six types (types I–VI), in which type II-A (CRISPR-Cas9), type V-A (CRISPR-Cas12a or Cpf1), and (CRISPR-Cas12b or C2c1) have been most widely studied (Adli, 2018; Yao et al., 2018a). More than 10 different CRISPR/Cas proteins have been repurposed for genome editing. Among them, some of the most recently discovered Cas proteins are hotspots for research, such as the Cas12a proteins from *Acidaminococcus* sp. (AsCas12a) and Lachnospiraceae bacteria (LbCas12a). Beyond Cas proteins, optimization of CRISPR systems has been thoroughly studied, including sgRNA design, cell enrichment, editing conditions, *etc*. With the rapid development and progress of gene editing technology, CRISPR systems have been shown to be powerful and highly efficient gene-editing tools in various fields. Through numerous experiments in model and non-model organisms (Oh et al., 2010), these systems have been utilized to reveal cancer mechanisms (Sottnik et al., 2021), define gene function and phenotypes (Johansen et al., 2017), and treat human diseases (Torre et al., 2021).

As to traditional editing tools, zinc finger nucleases and transcription activator-like effector nucleases (TALENs) have overwhelmingly contributed to developments in biomedical research and application (Urnov et al., 2005). Their application is greatly limited, however, due to limitations such as high cost, low efficiency, and low throughput targeting (Batool et al., 2021). In contrast, the CRISPR technology has some unique advantages, including targeted editing of multiple genomic sites (Zhang and Showalter, 2020), fast generation of mutants (Zhang and Showalter, 2020), and accessible sgRNA design (Xu et al., 2020b). These advantages have led to a surge in CRISPR applications in various fields, such as agriculture (Zheng et al., 2019), animal husbandry (Liu et al., 2020b), chemical fields (Liu et al., 2021b), materiology (Demirer et al., 2021), *etc*. Although the framework of the structures and functions of CRISPR/Cas systems has been built, there are still several challenges in this system (Wang et al., 2016), including off-target effects (Coelho et al., 2020), variable efficiency (Jin et al., 2020), requirement of PAM and sgRNA (Heussler et al., 2015; Cameron et al., 2017), and inactive mutants (Ren et al., 2019). This review proposes some strategies to overcome these issues by reducing off-target effects, improving the repair efficiency of the homology-directed repair (HDR) pathway, choosing the optimal delivery system, and utilizing variants of Cas proteins. Additionally, regulation of nuclease-dead mutants of Cas9, anti-CRISPR (Acrs) protein application, and enrichment of cells and sgRNA may be effective strategies for the efficacy of CRISPR/Cas systems.

## 2 Strategies for Reducing Off-Target Effects

Presently, off-target effects in CRISPR/Cas systems are a major issue for gene editing. Whether the Cas protein is off- or on-target to a PAM site is mainly determined by the sgRNA, Cas proteins, ribonucleoprotein (RNP) concentration, as well as other factors, such as editing temperature and action time. The off-target cleavage of CRISPR/Cas systems often originates from the unsuccessful design or modification of gRNA, low specificity of Cas proteins, or excessive and prolonged expression of CRISPR/Cas9. Accordingly, various strategies are proposed to overcome these issues. Additionally, methods for sgRNA selection with off-target predictions have been established, such as PEM-seq (Yin et al., 2019b), CRISPR-PLANT v2 (Minkenberg et al., 2019), and CRISPR-GE (Xie et al., 2017), which avoid a waste of manpower and material resources and improve editing efficiency.

### 2.1 Reasonable Design and Modification of sgRNA

**FIGURE 1:**
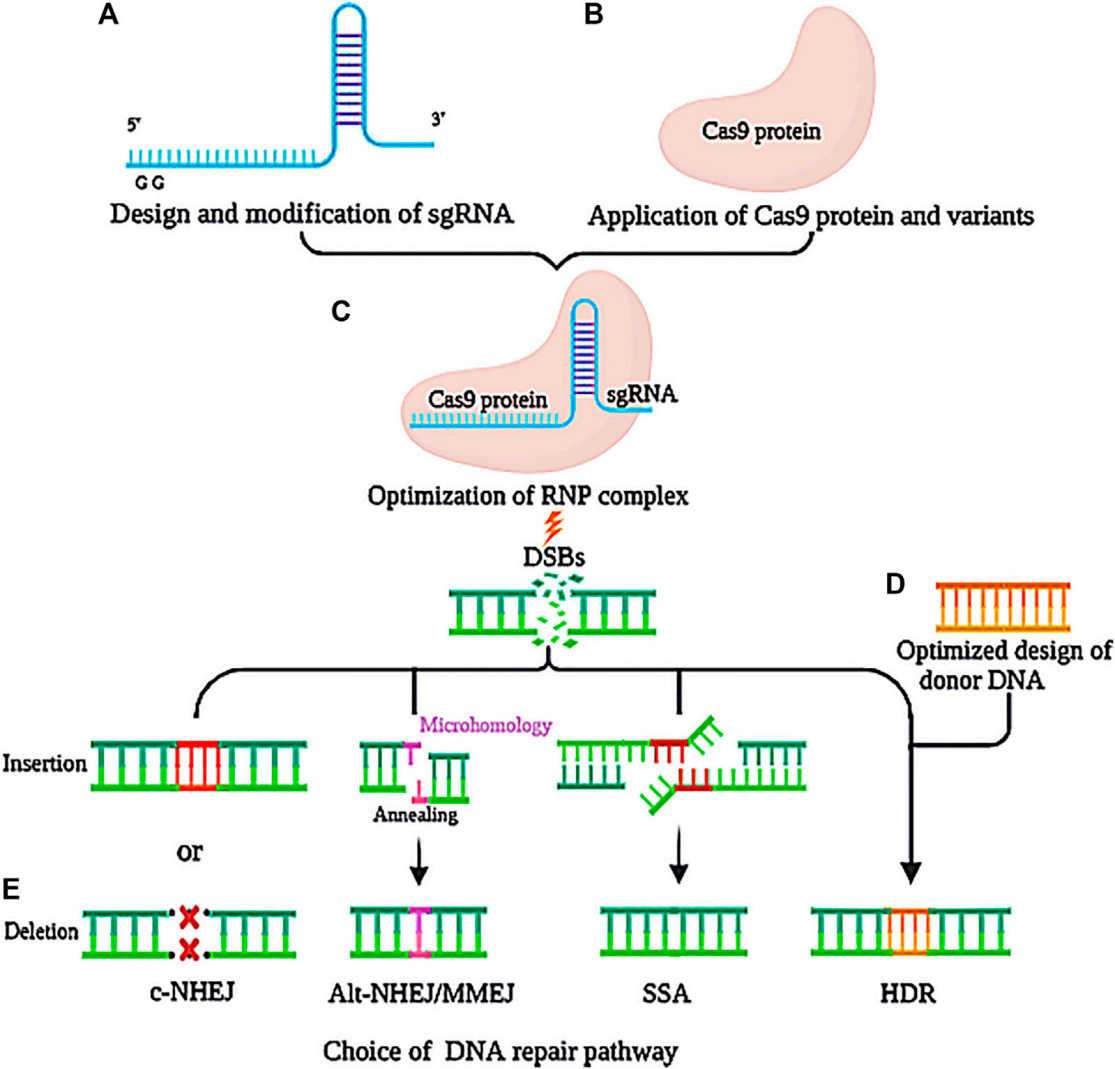
Optimization strategies of different steps of the CRISPR/Cas system. **(A)** The sgRNA sequence was optimally designed and modified by truncation or addition of 3’ or 5’ end of sgRNA, DNA-RNA chimera, etc. **(B)** Cas9 protein was optimized for concentration, temperature, and time, as well as application of variants. **(C)** Optimization of the RNP complex was conducted for proportion, function conditions, and transformation methods. **(D)** Donor DNA was optimized for design of the DNA template, proximity to CRISPR components, and choice of high-efficient delivery system. **(E)** DNA repair pathway was optimized with inhibition of the NHEJ pathway, enhancement of the HDR pathway, and modification of other pathways.

In CRISPR/Cas systems, the binding of sgRNA to the PAM site is a critical step in gene editing (Figure 1A). An unsuccessful design of sgRNA will result in lower specificity and higher miss rate (Doench et al., 2016). To avoid this, sgRNA must be accurately designed using computational tools (Liu et al., 2020a), such as CRISPR-P 2.0 (Liu et al., 2017a), E-CRISP (Heigwer et al., 2014), and CasFinder (Abby et al., 2014). On the basis of rational design, further modification of sgRNA can improve the specificity of RNA-guided Cas9 by truncation or addition of nucleotides to the 5′ or 3′ end (Pattanayak et al., 2013; Lin et al., 2014b). The 5′ end-truncated sgRNAs (2-3 bp) considerably reduce off-target mutations, but with the same on-target mutation efficiency as the full-length sequence (Fu et al., 2014). By decreasing the binding affinity of the sgRNA, the binding stringency of Cas9 to the target sequence was increased, and the off-target effect was reduced (Fu et al., 2014). Since truncated sgRNAs can reduce the off-target effect of paired Cas9 nickases without compromising the efficiency of on-target genome editing, their combination results in a much greater target specificity (Guilinger et al., 2014). Contrarily, the 3′ end-truncated sgRNA or 5′ end-added sgRNA (-GG) can decrease the on-target activity. Meanwhile, if they consist of 16 nucleotides or fewer, truncated sgRNAs exhibit lower or undetectable activity compared to matched full-length sgRNAs. Thus, at least 17-nucleotide sgRNAs are required for the CRISPR/Cas9 system to be active during gene editing. Due to the disadvantages of the traditional enzymatic preparation of sgRNAs, such as complexity, time consumption, and safety concerns, the direct chemical synthesis of sgRNAs has been widely accepted, with high sgRNA stability and low off-target effect. Recently, a potential strategy has been reported to reduce off-target editing by DNA–RNA chimera (Yin et al., 2018a). Using the Cas9–sgRNA complex as a guide, the 5′- and 3′-DNA-replaced crRNA enables more efficient genome editing—for example, replacing the crRNA with 10 DNA nucleotides could provide the same level of off-target site indel formation as the truncated sgRNA. Additionally, the synthesis cost of DNA bases is much lower (10-fold cheaper) than that of native crRNA. In light of this, the DNA–RNA chimera could provide a novel approach to reduce the cost and off-target effect of CRISPR/Cas systems.

### 2.2 Cas Variant Application

So far, several highly specific Cas9 variants have been identified, including eSpCas9 (Slaymaker et al., 2016), SpCas9-HF1 (Kleinstiver et al., 2016), HypaCas9 (Chen et al., 2017), xCas9 (Legut et al., 2020), Sniper-Cas9 (Lee et al., 2018), evo Cas9 (evolved Cas9) (Casini et al., 2018), HiFiCas9 (Vakulskas et al., 2018), and HeFSpCas9 (Kulcsar et al., 2017). The main mechanism and characteristics of each variant are comprehensively summarized in Table 1. Among these mechanisms, the most common cause of alteration in Cas9 function is amino acid substitution of the critical domain. Due to the minimum binding energy required to introduce DSBs into the genome, non-specific interactions between Cas9 and target DNA were reduced by decreasing the excess energy of wild-type SpCas9. As shown in Table 1, although they exhibit greater target specificity, each variant has its own limitations, such as low activity (Slaymaker et al., 2016), scope limitation (Kleinstiver et al., 2016), strict dependency on a PAM site (Legut et al., 2020), *etc*. Future studies should be conducted to increase the efficiency of genome editing using Cas9 variants. For a given target sequence, the optimal variant should be selected based on a comparison of activity, specificity, and PAM compatibility. By comparing 13 SpCas9 variants, the results demonstrated that the overall activity order of high-fidelity variants could be ranked as SpCas9 ≥ Sniper-Cas9 > eSpCas9 (1.1) > SpCas9-HF1 > HypaCas9 ≈ xCas9 > evoCas9, whereas their overall specificity could be ranked as evoCas9 > HypaCas9 ≥ SpCas9-HF1 ≈ eSpCas9 (1.1) > xCas9 > Sniper-Cas9 > SpCas9 (Kim et al., 2020b). Using established computational models, these results provide guidance for the selection of Cas9 variants and offer a more effective exploration of variants for future research (Figure 1B).

**TABLE 1:** The features of different Cas protein variants.

Cas variants	Description	Mechanisms	Target sequence	Average indel frequency	Advantages	Limitation	Reference
xCas9	Generation of xCas9 by “phage-assisted continuous evolution (PACE)” method	Closing to PAM or the DNA-sgRNA interface refines the DNA-RNA contact region	Refer to the three lentiviral libraries (Kim et al., 2020a)	32%	Improve the target specificity and extend the target range, present a higher DNA specificity and lower off-target activity	Profoundly diminished of xCas9 activity at target sites with NGH PAM	Nishimasu et al. (2018)
SpCas9-HF1	The quadruple substitution variant (N497A/R661A/Q695A/Q926A) of wild-type SpCas9	Reduce the rate of DNA cleavage but have no effect on the rate of DNA reversion and release	Refer to the three lentiviral libraries (Kim et al., 2020b)	34%	A high-fidelity variant retains on-target activities comparable to wild-type SpCas9 with >85% of sgRNAs	The unclear mechanism of target discrimination and fidelity needs to be further improved	Kleinstiver et al. (2016)
eSpCas9	SpCas9 mutants consisting of individual alanine substitutions at 32 positively charged residues within the nt-groove	Neutralization of positively charged residues within this non-target strand and then weaken non-target strand binding and encourage re-hybridization between the target and non-target DNA strands	Refer to the three lentiviral libraries (Kim et al., 2020a)	40%	Decrease the off-target activities and maintain efficient on-target editing	The unclear mechanism of target discrimination and fidelity needs to be further improved	Slaymaker et al. (2016)
HypaCas9	The quadruple substitution variant (N692A/M694A/Q695A/H698A) of wild-type SpCas9	The quadruple substitutions in the REC3 domain of wild-type SpCas9	Refer to the three lentiviral libraries (Kim et al., 2020b)	30%	Higher genome-wide fidelity without affecting the on-target genome editing	Not mentioned	Chen et al. (2017)
Cas9n	Inactivating of HNH or RuvC nuclease domains	Use dual-RNAs for site-specific DNA cleavage	Two human genes: C4BPB and CCR5	75 and 60%	Greater target specificity	Rational design of sgRNAs on the plus and minus strands within a limited distance	Trevino and Zhang (2014)
Sniper-Cas9	The quadruple substitution variant (F539S/M763I/K890N) of wild-type SpCas9	Weakening non-specific interactions between RNP and its substrate DNA	Refer to the three lentiviral libraries (Kim et al., 2020a)	46%	Retain WT-level on-target activity with diminished off-target effect	Not mentioned	Lee et al. (2018)
evoCas9	The quadruple substitution variant (M495V/Y515N/K526E/R661Q) of wild-type SpCas9	Weakening non-specific interactions between RNP and its substrate DNA	Refer to the three lentiviral libraries (Kim et al., 2020b)	15%	Retain WT level on-target activity with diminished off-target effect	Not mentioned	Casini et al. (2018)
HiFiCas9	The quadruple substitution variant (R691A) of wild-type SpCas9	Weakening non-specific interactions between RNP and its substrate DNA	Five human genes: HBB, IL2RG, CCR5, HEXB, and TRAC	Similar to WT Cas9	Retain WT level on-target activity with diminished off-target effect	Not mentioned	Vakulskas et al. (2018)
HeFSpCas9	The quadruple substitution variant (N497A/R661A/K846A/Q926A/K1003A/R1060A) of wild-type SpCas9	Combinations of mutation domain from both eSpCas9 and SpCas9-HF1	Not shown	Not shown	Retain WT level on-target activity with diminished off-target effect	Not mention	Kulcsar et al. (2017)

### 2.3 Determination of the Optimal RNP Concentration

**FIGURE 2:**
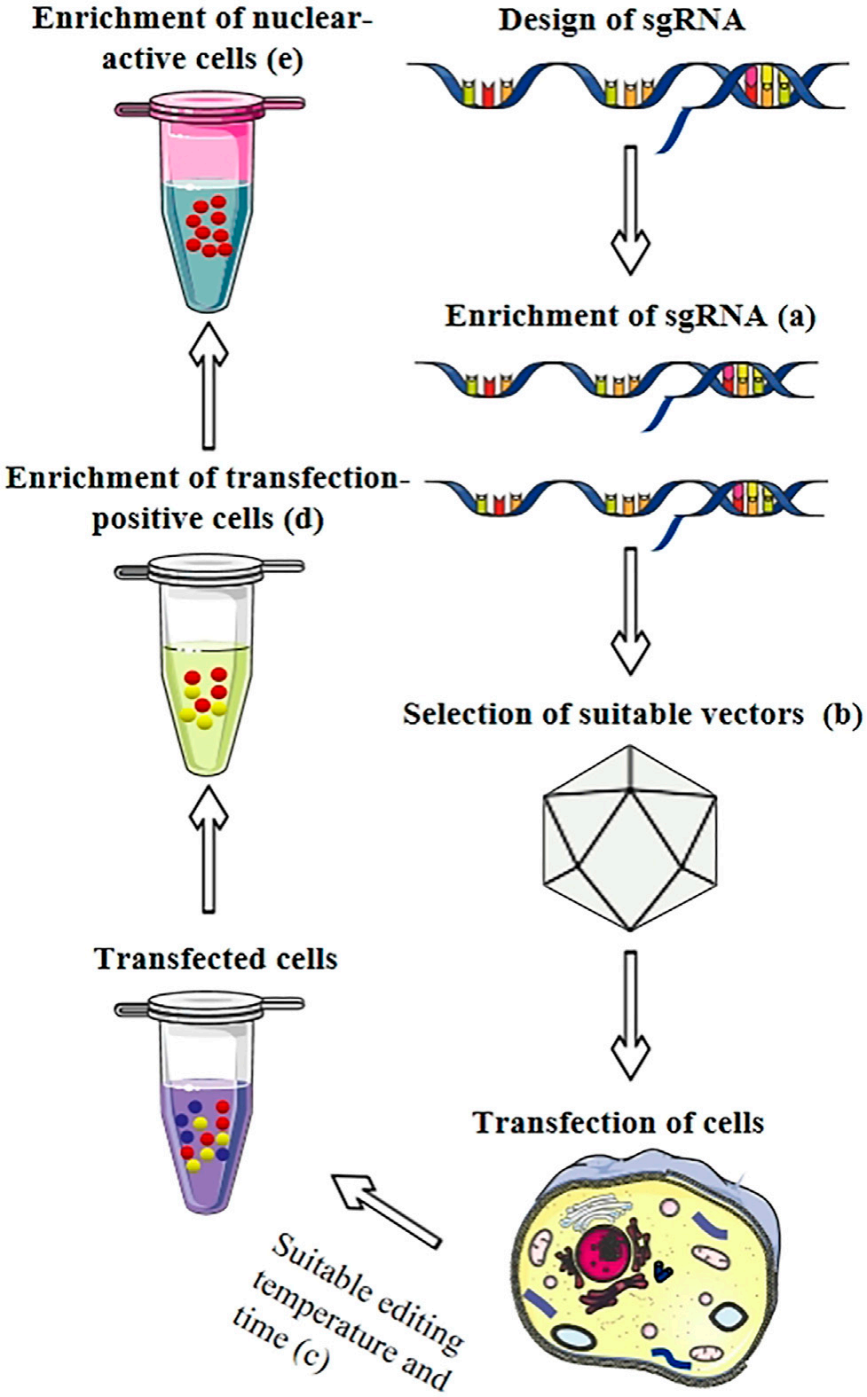
Enrichment strategies for sgRNA and mutants in CRISPR/Cas systems. **(A)** The sgRNA was enriched with by PCR or CRISPR. **(B)** The optimal vector was selected according to the different host cell and other factors. **(C)** Optimization of editing time and temperature was conducted through application of nuclease-dead mutants of Cas9 and anti-CRISPR proteins, heat stress method. **(D)** Transfection-positive cells were enriched based on fluorescent proteins, antibiotic-resistance genes, and cell-surface antigens. **(E)** Nuclear-active cells were enriched using NHEJ-based and SSA-based surrogate reporters.

In general, the specificity and the activity of enzymes are often highly dependent on reaction conditions. RNP delivery produces at least twofold more colonies than plasmid transfection does (Kim et al., 2014). In the CRISPR/Cas9 system, RNP concentration plays a decisive role in both specificity and activity. After delivery to cells, RNPs almost immediately cleave chromosomal DNA and then degrade rapidly. With a high RNP concentration, the off-target effects of a CRISPR/Cas system may be amplified (Figure 1C). Meanwhile, a low RNP concentration leads to a reduction of on-target cleavage efficiency. Therefore, a suitable concentration of RNP is of paramount importance to minimize nonspecific cleavage (Figure 1E). This can be achieved by using either low concentrations of plasmids or different promoters. The former method directly reduces RNP transcription, while the latter alters the 5′-untranslated region of the target sequence, ultimately affecting translation efficiency (Hsu et al., 2013). Therefore, extensive measurements should be performed with consideration of both Cas9 activity and specificity. Compared with typical RNP concentrations, on-target activity will inevitably be inhibited to some extent. By modifying Cas9 and sgRNA instead (Figure 2A), the intrinsic specificity of Cas9 can be improved without sacrificing cleavage efficiency.

### 2.4 Suitable Editing Time and Temperature

During gene editing, the efficacy, efficiency, and accuracy of CRISPR systems are often limited by temperature and time (Figure 2C). Studies have shown that a longer editing time of the Cas proteins in cells increases the off-target effects and negatively impacts outcomes (Ruan et al., 2017). Therefore, reducing the action time or overexpression of Cas proteins enhances the efficiency of gene editing. Early reports have utilized tissue culture-free systems (Manghwar et al., 2020), DNA-free systems (Kim et al., 2020a), and transient systems (Canto, 2016) to overcome these limitations. Now, however, anti-CRISPR proteins and nuclease-dead Cas proteins can be used to address these limitations. Temperature has been shown to affect Cas protein activity, but the findings are inconsistent. Hoyland-Kroghsbo *et al*. (2018) reported that a lower temperature is more effective than a higher one in *P. aeruginosa* PA14 due to the greater stability of the CRISPR/Cas complex. Additionally, low temperatures may enhance interference and adaptation by increasing the annealing efficiency of the crRNA to its target. Another report has shown that exposure to heat stress results in a greater amount of targeted mutations than with exposure to standard temperature (22°C) (LeBlanc et al., 2018). This is likely due to the fact that the activities of Cas9 and Cas12a at 37 and 34°C, respectively, are higher than at 28°C, and the expression level of sgRNA is raised at 39°C (Xiang et al., 2017). The mechanism of the effect of temperature on CRISPR/Cas systems is still unclear and should be further elucidated.

### 2.5 Application of Anti-CRISPR Proteins

In the course of long-term antagonism between bacteria and phages, the phages have evolved Acr proteins to evade CRISPR/Cas-mediated immunity. Up to now, a total of 44 Acr proteins have been identified and named (Zhang et al., 2019a). Within the CRISPR/Cas system subtypes, these Acr proteins are present in types I, II, and V, but not in other subtypes—for instance, FnCas9 (Green and Hu, 2017) and C2c2 (Zhang et al., 2019a) Acr proteins were not found in the subtype II-B CRISPR/Cas9 system and the type VI CRISPR/Cas13a system, respectively. In view of this, the identification and characterization of a novel Acr is a key focus for future studies. As natural inhibitors, Acrs protect the host genome from destruction by inhibiting Cas nuclease activity. This inhibition can be achieved through the following three mechanisms: (1) inhibition of Cas9 binding to DNA (Malone et al., 2020), (2) interference of Cas9 binding to gRNA (Harrington et al., 2017), and (3) blocking the activity of Cas9 (Harrington et al., 2017). Currently, only AcrIIA2 and AcrIIA4 have been utilized as tools to reduce off-target effects. Through competition with the PAM site and/or other Cas9 sequences, these Acr proteins block the cleavage activity of Cas9, preventing the excessive and prolonged expression of CRISPR/Cas9 and thus decreasing off-target effects (Hoffmann et al., 2019; Liu et al., 2019). Acrs appear to be a new agent to improve the accuracy and safety of CRISPR-based therapies. Other special functions of Acr proteins also deserve attention—for example, AcrII-C3 can precisely regulate gene expression with dCas9-based tools, which is very helpful for the development of versatile genome engineering modulators (Liu et al., 2018c). The optogenetic-controlled AcrIIA4 enables light-mediated genome and epigenome editing. By inserting the AsLOV2 domain into the most C-terminal loop of AcrIIA4, the protein can switch the CRISPR-Cas9 activity according to light/dark conditions (Bubeck et al., 2018). The mechanisms of other Acrs, such as AcrIIA5-10 and AcrVA2-3, have yet to be described (Zhang et al., 2019a).

## 3 Strategies to Improve the Efficiency of the HDR Repair Pathway

After Cas9 nuclease cleavage, DSBs can be repaired in a host through at least one of two different pathways: nonhomologous end joining (NHEJ)/canonical NHEJ (c-NHEJ) and HDR (Ghezraoui et al., 2014; Sander and Joung, 2014). While c-NHEJ is the predominant approach, due to its speed and high efficiency, it is also prone to error because of leading uncertain inserts or deletions (indels). Indels contribute to the generation of a targeted knockout during cell repair (Pawelczak et al., 2018). The HDR pathway enables accurate genome editing in a variety of manners, such as gene knock-in, knockout, replacement, and point mutations (Platt et al., 2014; Zuo et al., 2017; Vakulskas et al., 2018; Lu et al., 2020). However, due to competition with the NHEJ pathway, the HDR pathway tends to be less efficient (Liu et al., 2018a). Given this, different approaches have been established to improve the repair efficiency of the HDR pathway, including inhibition of the NHEJ pathway (Maruyama et al., 2015), regulation of HDR-related factors (Paulsen et al., 2017), cell cycle synchronization (Ferrari et al., 2020), optimal design of the donor DNA template (Renaud et al., 2016), and optimizing the proximity of the CRISPR component and donor DNA template (Ma et al., 2017a). These strategies are discussed in detail in the following paragraphs.

### 3.1 Inhibition of Nonhomologous End Joining Pathway

In theory, because of the competition between the two repair pathways, the efficiency of the HDR pathway can be boosted by inhibiting key factors of the NHEJ pathway. Among different inhibitors of the NHEJ pathway, SCR7 is a key factor that interferes with the affinity of DNA ligase IV to DSBs (Srivastava et al., 2012; Chu et al., 2015; Maruyama et al., 2015; Li et al., 2017; Shao et al., 2017; Hu et al., 2018). Maruyama *et al*. (2015) reported that using SCR7 increased the efficiency of HDR-mediated genome editing by up to 19-fold with the most significant enhancement effect, primarily due to co-injection of the CRISPR-Cas9 constructs with SCR7 into zygotes rather than other cells. The combination of SCR7 with other factors could significantly improve the efficiency of the HDR pathway by either downregulating KU expression (Chu et al., 2015), optimizing the donor template (Hu et al., 2018), or upregulating Rad52 expression and other small molecules (Li et al., 2017; Shao et al., 2017). Among these methods, the efficiency of the HDR pathway using Rad52 combined with SCR7 is the highest, reaching up to 40% (Shao et al., 2017). However, the effect of SCR7 in enhancing the HDR pathway remains controversial at present (Greco et al., 2016), with some reporting that embryonic stem cells tend to occur intrinsically HDR incident, suggesting that the effect of SCR7 is likely uncertain (Yang et al., 2020). Therefore, the effect of SCR7 in increasing HDR efficiency needs to be further explored. Additionally, the use of SCR7 should be extended to other fields as well instead of being limited to human- and mammalian-related diseases only, whether used alone or in combination with other medicines (Manjunath et al., 2021).

Aside from SCR7, other approaches also improved the efficiency of the HDR pathway—for instance, by downregulating KU protein expression *via* siRNA silencing, the frequency of the HDR pathway can be increased at least slightly (Li et al., 2018a). This method raised the affinity of downstream NHEJ enzymatic components by attaching to DNA termini upstream of the NHEJ pathway (Mateos-Gomez et al., 2017). The combination of multiple inhibitors can further increase the inhibitory effect on the NHEJ pathway—for example, M3814 combined with trichostatin A inhibits the NHEJ pathway 3-fold (Fu et al., 2021). NU7441 and KU-0060648, inhibitors of a key NHEJ pathway factor, DNA-PK, caused a 2-fold increase in HDR efficiency in HEK-293T cells (Robert et al., 2015). Due to the fact that the NHEJ pathway is crucial for the stabilization of the genome, excessive inhibition of the NHEJ pathway may eventually lead to the accumulation of unrepaired DSBs in cells, inducing cell death or embryonic lethality (Beumer et al., 2013). Therefore, it is worth noting that the safety of these inhibitors needs to be carefully evaluated in future work.

### 3.2 Regulation of HDR-Related Factors

Alternatively, compared with inhibition of the NHEJ pathway, direct regulation of HDR-related factors can result in more precise editing and improve the efficiency of the HDR pathway. Several HDR-related factors have been well studied, including Rad51 (Ma et al., 2020), Rad52 (Shao et al., 2017), C-terminal-binding protein interacting protein (CtIP) (Charpentier et al., 2018), and RS-1 (Song et al., 2016). All of these factors enhance a link in the HDR pathway, thereby boosting repair efficiency. Among these, the overexpression of Rad proteins and the application of CtIP result in superior outcomes. In the HDR pathway, Rad51 proteins search for a DNA donor template to perform reconstitution through the formation of filaments on the DNA (Symington, 2014). As a back-up for Rad51, Rad52 is involved in the formation of Rad51 (Lok and Powell, 2012). When Rad52 fuses with any other factor or factors, HDR efficiency can be boosted at least 2- to 7-folds and sometimes much more (Paulsen et al., 2017; Shao et al., 2017; Tran et al., 2019)—for example, Rad52 fused with Cas9 yielded an approximately 3-fold increase in the efficiency of the HDR pathway, while Rad52 combined with SCR7 increased the HDR efficiency by about 40% (Shao et al., 2017). In the early stage of the HDR pathway, a key protein, CtIP, initiates the resection process and then creates 3′ single-stranded overhangs with exonuclease 1 and bloom syndrome protein complex (Symington, 2014). When combined with Cas9 or the MS2 system, CtIP can shift the ratio of the HDR/NHEJ pathway activities by a factor of 14.9 (Tran et al., 2019). A minimal N-terminal fragment of CtIP can also be used as an HDR enhancer, which is sufficient to stimulate the HDR pathway and improve repair efficiency by at least 2-fold (Charpentier et al., 2018). Other small molecules, such as L755507 and resveratrol, can also increase HDR efficiency 2- to 3-fold (Li et al., 2017). Almost all key factors of the HDR pathway were upregulated after treatment with the small molecules mentioned above, but a high concentration of resveratrol (more than 50 μM) resulted in severe cytotoxicity, significantly reducing cell viability and slightly upregulating the expression of the NHEJ factor. Therefore, the application of these factors needs to be further explored to improve the efficiency of the HDR pathway.

The selection of the NHEJ or HDR pathway plays a pivotal role in DNA repair. As an inhibitor of the HDR pathway, the tumor suppressor p53-binding protein 1 (53BP1) blocks DNA end resection and simultaneously inhibits BRCA1 recruitment to DSB sites (Panier and Boulton, 2014; Zimmermann and de Lange, 2014). By means of fusing, inhibiting, and binding 53BP1, HDR repair frequency can be increased from 20 to 86% (Paulsen et al., 2017; Canny et al., 2018; Jayavaradhan et al., 2019). Compared with a control, the correction frequency of the HDR pathway increased by nearly 20% when RAD52 was ectopically co-expressed with dominant-negative 53BP1 (dn53BP1) (Paulsen et al., 2017). Interestingly, dn53BP1 itself did not improve HDR efficiency unless combined with Rad52, suggesting that this fragment may not effectively promote the HDR pathway. To avoid the unwanted effects of global NHEJ inhibition, the fusion of DN1S and dn53BP1 significantly blocked NHEJ events locally while improving the correction frequency of HDR up to 86% (Jayavaradhan et al., 2019). This effect was likely due to the restrictively suppressive effect of dn53BP1 around the DSB site, which makes the CRISPR-Cas9-DN1S system a far more effective and stable approach in clinical treatments with high HDR frequency. Inhibition of 53BP1 is another indispensable strategy for regulating different repair pathways—for instance, utilization of 53BP1 inhibitor (i53) resulted in up to a 5.6-fold gene conversion and demonstrated an effective impact on the conversion mediated by single-stranded oligodeoxynucleotides (ssODN) compared to canonical modality double-stranded DNA (dsDNA) donors (Canny et al., 2018). However, the unknown toxicities or adverse incidents of i53 need to be carefully considered before its use. Moreover, the direct binding of related proteins to 53BP1 is a new target for enhancing the HDR pathway. A typical example is the TIRR protein, which acts similar to i53 and provides strong suppression by binding directly to 53BP1 (Anuchina et al., 2020). Since the function of TIRR is unclear, more studies should focus on its mechanism and the potential effects on the HDR pathway. TIRR may hold promise as a new target for enhancing the HDR pathway in genome editing.

### 3.3 Synchronization of Cas9 Activity and HDR-Active Cell Cycle

Since HDR repair activity is restricted to the S and G2 phases of the cell cycle, synchronizing cells in these phases can significantly enhance the repair activity. In terms of a single aspect of cell cycle synchronization, chemicals that maintain the cell cycle in the S and G2 phases containing nocodazole (Yiangou et al., 2019), ABT-751 (Yang et al., 2016), and RO-3306 (Sunada et al., 2021) have been commonly used in CRISPR systems and increased the HDR efficiency by a factor of 1.38–6 (Lin et al., 2014a; Yang et al., 2016; Wienert et al., 2020). ABT-751 and nocodazole arrest the cells in the G2/M phase by inhibiting microtubule polymerization (Vasquez et al., 1997; Hande et al., 2006). Meanwhile, RO-3306 can transiently arrest cells at the transition from G2 to M phase by inhibiting the CDK1 function, enriching the number of cells in the S and G2 phases (Vassilev, 2006). Recently, Lomova *et al*. reported that the transient suppression of Cas9 activity and synchronization of the HDR-active cell cycle may have a prominent effect on the HDR pathway. RO-3306 combined with Cas9, which nuclease activity is reduced in the G1 phase, can improve the HDR/NHEJ ratio 20-fold, thereby limiting unnecessary NHEJ events (Lomova et al., 2019). In addition, the timed delivery of pre-assembled Cas9 RNP and chemical synchronization agents can also enhance the HDR rates by up to 38% (Lin et al., 2014a). Thus, synchronizing the cell cycle paired with controlled timing of Cas9 activity might be more practical and safer than synchronizing the cell cycle alone. In conclusion, more efficient synchronization agents for *in vitro* application need to be further studied. Agents with lower toxicity should also be explored for *in vivo* application in subsequent research. More importantly, there is no doubt that the combination of multiple technologies, such as controlled timing of Cas9 activity and cell cycle synchronization, will result in better outcomes.

### 3.4 Increasing the Proximity of CRISPR Components to the Donor DNA Template

At the time of DNA cleavage, if the donor DNA template is in closer proximity to the CRISPR components or has a higher concentration in the nucleus, the efficiency of the HDR pathway can be significantly increased (Devkota, 2018). Based on this hypothesis, many studies have demonstrated its feasibility and potential value in clinical applications (Liang et al., 2017). By attaching the donor DNA template to modified sgRNA, a S1mplex strategy improves the enhancement of the HDR pathway. In this system, the modified S1m-sgRNA adds an aptamer, which binds the streptavidin protein. Biotinylated ssODN linked to the streptavidin then increases proximity. Through this powerful S1mplex strategy, the ratio of HDR increased 18-fold compared with the unlinked components (Carlson-Stevermer et al., 2017). By virtue of the affinity between avidin and biotin, Ma *et al*. devised a Cas9 variant that was fused to avidin *via* a flexible linker and bridged with biotin-modified ssDNA to increase the proximity. This system achieved ∼20% HDR frequency in mouse embryos (Ma et al., 2017a). HUH endonuclease is a bridge that is also capable of forming robust covalent bindings with unmodified donor DNA templates (Lovendahl et al., 2017). Utilizing this convenient technique could create a stable Cas9 RNP–ssODN complex (Aird et al., 2018). Additionally, Natasa *et al*. linked ssODN to Cas9 through SNAP-tag technology, allowing O6-benzylguanine-labeled ssODN to covalently bind to SNAP-tag fusion proteins (Savic et al., 2018). Both SNAP-tag and HUH-tag enable the spatio-temporal co-localization of the donor templates at DSBs, thus achieving 24- and 30-fold enhancement of HDR efficiency, respectively. In addition to ssODN attachment to sgRNA in the S1mplex system, other experiments use a variety of ways to attach ssODN to Cas9 protein. Among them, the HUH-tag strategy displayed a more promising prospect due to the superior ease of use and lower cost associated with modifying donor DNA. As noted above, Cas9 RNP complexes can connect with donor DNA templates through a variety of chemical modifications, all of which significantly enhance the transient expression of the HDR pathway. Furthermore, Cas9 and sgRNA delivered in the RNP format also exhibited a lower frequency of unwanted mutations and off-target effects (Svitashev et al., 2016), suggesting that the RNP format may be a promising approach in the broad field of gene editing.

### 3.5 Selection of the Donor DNA Template

To a considerable extent, the repair efficiency of the HDR pathway depends on the selection of donor DNA, including DNA modality, length, and flanking homologous sequences (Song and Stieger, 2017; Renaud et al., 2016). Generally, the modality of a DNA template can be divided into three forms: plasmid, ssODN, and linear dsDNA (Figure 1D). As the most common form of genetic material, circular plasmids are widely used in gene editing but will not be discussed in this review (Bosch et al., 2020; Sondergaard et al., 2020; Yoshimi et al., 2021). Compared with other donor DNA templates, ssODNs have the advantages of ease of design, lower time investment, less illegitimate random integration (introducing less than 200 nucleotides), and site-specific modification for precise editing (Yang et al., 2013; Miura et al., 2018). By comparing the modification efficiency of CRISPR/mRNA and CRISPR/nuclease for a target gene, the results have shown that the 36-nt length of ssODN with the CRISPR/nuclease form achieved the optimal condition for modification of the target gene, with a slight advantage over the CRISPR/mRNA approach (Kumita et al., 2019). Currently, ssODNs have become a routine editing tool both *in vitro* and *in vivo*, especially in multigene knock-in experiments (Yoshimi et al., 2016; Miura et al., 2018; Lim et al., 2020). For large sequence DNA modifications, linear dsDNA templates (up to 11 kb) were commonly used for CRISPR systems with homology arms of 500–800 bp (Yang et al., 2013). The targeted integration of linearized dsDNA–CRISPR can increase the knock-in efficiency 12-fold by injecting PCR-amplified donor DNA, Cas9 mRNA, and sgRNA (Yao et al., 2018b). Therefore, optimal editing outcomes can be obtained by selecting the suitable donor DNA modality according to experimental requirements.

## 4 Selection of a Highly Efficient Delivery System

So far, numerous delivery systems to deliver drugs and genes have been developed (Figure 2B). In this section, we have selected the current delivery systems with high delivery efficiency, potential for development, high biological safety, and strong tissue specificity for overview. According to their biological characteristics, they can be classified as either bioactive or abiotic. In bioactive systems, common CRISPR delivery systems contain viral vectors (Jarrett et al., 2018; Boucher et al., 2020; Lee et al., 2021), extracellular vehicles (Yao et al., 2021), cell-penetrating peptides (CPPs) (Ramakrishna et al., 2014b), or lipid nanoparticles (Cheng et al., 2020). In abiotic systems, gold nanomaterials (Wang et al., 2018), polymers (Lv et al., 2018), and graphene oxide (Yue et al., 2018) had a better effect on CRISPR system delivery. Several prominent reviews have comprehensively described the mechanisms, efficiency, challenges, and future directions for each of these systems (Yin et al., 2017; Li et al., 2018b; Glass et al., 2018; Wilbie et al., 2019; Zhang et al., 2021). The current status of these delivery systems will be exposited in the following paragraphs. Conventional physical delivery methods, such as electroporation (Shi et al., 2018), microfluidics (DiTommaso et al., 2018), and microinjection (Xu, 2019), possess unique advantages, including high local tissue transfection efficiency and extensive cellular adaptability (Mashel et al., 2020). They are not good candidates for this review, however, as they can also cause cell damage and potentially substantial cell death. Therefore, we did not repeat their descriptions in this article.

### 4.1 Bioactive Delivery Systems

#### 4.1.1 Viral Vectors

In recent years, viruses have been represented as an essential and powerful tool for CRISPR due to their efficient gene delivery and long-term stable transgenic expression (Heckl et al., 2014). The most commonly utilized viral vectors are derived from adeno-associated virus (AAV) (Jarrett et al., 2018), lentivirus (LV) (Lee et al., 2021), adenovirus (Boucher et al., 2020), and baculovirus (Yin et al., 2021). These viral vectors have been widely used to deliver CRISPR/Cas9 elements for remedying genetic defects, like hearing loss (Omichi et al., 2019), neurological disorders (Pena et al., 2020), muscular dystrophies (Crudele and Chamberlain, 2019), and cystic fibrosis lung disease (Wold and Toth, 2013; Hart and Harrison, 2017). Several excellent reviews concerning different aspects of viral vectors for CRISPR-based genome editing have been published, covering topics such as viral mechanisms (Xu et al., 2019), viral vector application (Song et al., 2021), and viral vector progress (DiCarlo et al., 2017). Although highly efficient, viral vectors are presently hindered by their inherent disadvantages of carcinogenesis, insertion size limitation, immune response, genotoxicity, cytotoxicity, and difficulties of large-scale production (Matrai et al., 2010; Kotterman et al., 2015; Chen and Goncalves, 2016; Chen et al., 2020a; Shirley et al., 2020). These viral vectors have been improved in other aspects, such as pseudotyped LV and dual-AAV systems. When delivering cargo into cells, LVs need to interact with a cellular receptor to trigger the fusion of the viral envelope with the cell membrane. The envelope glycoprotein on the LV surface is exchanged with a heterologous glycoprotein in a process known as pseudotyping. Pseudotyped LVs consist of virus particles bearing glycoproteins derived from other enveloped viruses. Thus far, a variety of viral glycoproteins, including vesicular stomatitis virus (Liu et al., 2017c; Sena-Esteves and Gao, 2018; Rust et al., 2020), baboon endogenous retrovirus (Belot et al., 2019), and feline endogenous retrovirus (Zucchelli et al., 2017), have been incorporated into LVs to improve their infectivity and confer a more selective tropism. The versatile tropism of pseudotyped LVs has been utilized in the treatment of tumors (Lee et al., 2021) and gene modification (Gutierrez-Guerrero et al., 2020). AAV vectors are hindered by their relatively low packaging capacity (Wu et al., 2010), with a packaging range of no more than 5 kb, making them inappropriate for the delivery of larger Cas9 variants (Mali et al., 2013). To address this issue, dual-AAV systems have been explored, in which one encodes Cas9 and another encodes gRNA, resulting in a large target gene transfer (Zhi et al., 2022). It needs to be pointed out, however, that the disadvantages of this system limit its clinical application, such as low probability of delivering both viral vectors to the same cell and insufficient expression efficiency.

#### 4.1.2 Extracellular Vesicles

Whether *in vitro* or *in vivo*, extracellular vesicles (EVs) have been widely used to efficiently deliver genes or drugs (Choi et al., 2016; Montagna et al., 2018; Campbell et al., 2019; Mangeot et al., 2019; Gee et al., 2020). As natural cell-derived membrane vesicles, EVs serve the function of cell-to-cell communication with outstanding biocompatibility and immune-privileged characteristics. EVs are also hardly cleared by the immune system, avoiding the occurrence of hypersensitivity reactions (Zhang et al., 2014). Since EVs do not contain viral genomes, they have significant biosafety without the risk of endogenous virus recombination (Fuenmayor et al., 2017). Additionally, EVs transmit Cas9 with transient exposure, reducing the off-target chance triggered by Cas9 overexpression (Wu et al., 2014). All these advantages demonstrate an excellent potential for EVs as endogenous nano-vehicles in various fields. However, a major obstacle for EVs is the lack of robust tissue-specific delivery to specific cells. Targeted ligand modification on the surface of EVs is a promising avenue to ameliorate this weakness (Mathieu et al., 2019)—for instance, valency-controlled tetrahedral DNA nanostructures (TDNs) conjugated with DNA aptamers can be anchored on the EV surface *via* cholesterol, improving cell-specific delivery (Zhuang et al., 2020). The 3D tetrahedral steric superiority of TDN DNA aptamers can minimize lateral interactions among DNA, resulting in increased receptor–ligand binding and greatly enhancing tissue specificity. Compared with a control group, the TDN1-EVs-RNP group maximally restrained tumor growth in terms of tumor weight, volume, and percentage of tumor cells, demonstrating that the modified group accomplished a 2-fold increase in indel rate (up to 30%). Recently, EVs have been used in chimeric-antigen receptor (CAR) T-cell therapy to deliver CRISPR components to target cells precisely. By expressing chimeric-antigen receptors on vesicles derived from T cells, the anti-CD19-CAR-EVs preferentially accumulated in tumors compared to the liver, kidney, and other healthy tissues. Nevertheless, normal EVs were more evenly distributed throughout the body (Xu et al., 2020a). In addition to delivering CRISPR/Cas9 components, EVs also show great potential for drug delivery (Mateescu et al., 2017; Yang et al., 2018), anticancer therapy (Pascucci et al., 2014; Saari et al., 2015), and antigen delivery for vaccine development (Rabu et al., 2019).

#### 4.1.3 Lipid Nanoparticles

Lipid nanoparticles (LNP) as CRISPR delivery vehicles have attracted the interest of scientists (Yin et al., 2014; Kulkarni et al., 2019). They not only help CRISPR components cross cell membranes but also protect them from enzymatic degradation and immune responses (Liu et al., 2018b; Noll et al., 2018). Due to the advantages of excellent controlled release, targeting, and high stability, LNPs have been widely used as a CRISPR delivery vector for all kinds of cargo modality, such as plasmid DNA, mRNA, and RNP complexes (Li et al., 2018c; Li et al., 2019). Theoretically, endocytosis is considered to be the key to cell internalization for almost all common LNP materials. To improve tissue specificity and delivery efficacy, several new strategies have been reported in recent years. Firstly, based on the hypothesis that charge adjustment can mediate tissue-specific delivery, a new strategy termed selective organ targeting (SORT) has been established. By adding DOTAP (a permanently cationic lipid) and constantly regulating its proportion to the original composition of LNP, we can control the charge for tissue-specific delivery (Cheng et al., 2020). The results show that this SORT strategy can achieve high organ selectivity for CRISPR cargos delivered in the lung, spleen, liver, and other organs. Among these organs, delivery to hepatocytes has the highest specificity at 93%. Secondly, ultrasound has been reported to facilitate the delivery of CRISPR components (Shen et al., 2016; Yoon et al., 2017). Ultrasound at specific locations can cause microbubbles to create local membrane deformations and pore formation in response to acoustic energy (Taniyama et al., 2011; Zhou et al., 2012). LNP released by microbubbles can then be transferred directly into the cytoplasm by diffusion. The results show that LNP incorporated with microbubbles can effectively facilitate cargo to the target site for RNP delivery, and the editing efficiency of Cas9 RNP was improved by 71.6% (Ryu et al., 2020). Thirdly, under optimized synthetic conditions, microfluidic device-designed lipid nanoparticles achieved intracellular RNP delivery with 97% gene disruption and 23% base substitution without any apparent cytotoxicity (Suzuki et al., 2020). In short, optimizing the formulation of LNP or integrating other technologies into the delivery system will be a crucial direction for achieving tissue-specific and efficient systems. Lipid-based formulations, however, do have some disadvantages. Once nanoparticles pass through the surface of cells, they are typically encased within an endosome. The encased contents then enter the lysosomal pathway directly and are eventually degraded. Therefore, coating polymers on the LNP surface or developing other unique chemical modifications to facilitate cellular uptake and disrupting endosomal membranes are promising directions that could prompt endosomal escape and avoid detection by the immune system.

#### 4.1.4 Cell-Penetrating Peptides

As short stretches of amino acids, CPPs are polycationic, amphipathic, or non-polar in nature and possess an intrinsic ability to translocate across cell membranes (Suresh et al., 2017). Owing to the advantages of low cytotoxicity, better biological tolerance, less off-target effect, and no chemical reagent, CPPs have been exploited to deliver different cargos into cells *in vitro* and *in vivo* (Liu et al., 2014; Gagat et al., 2017). When delivering RNP complexes, CPPs conjugated with RNP to form CPPs–RNP, which can improve cellular uptake and/or fusion. However, few studies have been reported on CPP-mediated CRISPR component delivery at present. Moreover, both delivery efficiency and subsequent editing efficiency were usually at a low level of just 10–20% (Ramakrishna et al., 2014b; Yin et al., 2018b; Del’Guidice et al., 2018; Yin et al., 2019a). This likely stems from the indefinite mechanism of CPP internalization and requirement for extensive optimization for targeting each type of cargo and cell. As the major CPP cargo is trapped in endosomes, they end up being recycled or degraded in a targeted manner instead of releasing cargo to the specific destination. Thus, enhancing endosomal escape would be a potential approach to improve the efficiency of delivery and editing (LeCher et al., 2017).

### 4.2 Abiotic Delivery Systems

As an alternative, abiotic vectors may offer tantalizing possibilities for CRISPR/Cas9 delivery systems due to their low immunogenicity, larger delivery gene payload, ease of large-scale production (Li et al., 2015), and absence of endogenous virus recombination. Many excellent delivery systems with new properties have been established in various fields, such as gold nanomaterials (Wang et al., 2018), polymers (Lv et al., 2018), and other systems. The characteristics of each material are described in detail in the following sections.

#### 4.2.1 Gold Nanomaterials

Due to their tunable surface functionalization, non-toxic nature, favorable size, optical properties, biocompatibility, and photothermal effect, inorganic gold nanocarriers have proved to be a promising platform for systemic gene delivery (Ghosh et al., 2008; Ma et al., 2017b). They are mainly characterized by their photothermal effect and ease of functionalization for delivering CRISPR components. As photothermal transducers, gold nanomaterials can regulate the conditional control of Cas9 activity through different optical means (Nihongaki et al., 2015). In locally specific tissues, heat converted by the second near-infrared optical window (1,000 to 1,700 nm) induces endonuclear transformation of the heat-shock factor (HSF) from an inactive monomer to an active trimer. Under the action of active HSF, the combined transfection of a cationic polymer-coated Au nanorod, Cas9 plasmid, and a heat-inducible promoter HSP70 can result in 90% GFP-positive cells, which is much higher than that of Lipofectamine 2000 or 25-kDa polyethyleneimine (Chen et al., 2020b). In the LACM system, the protective DNA-modified gold nanorod hybridizes with the target binding domain of sgRNA to protect sgRNA. Upon NIR laser irradiation, heat subsequently denatures the hybridized DNA and sgRNA, accomplishing the controlled release of sgRNA into cells (Peng et al., 2020). Thus, gold nanomaterials act as an optogenetic switch to regulate the expression and activity of Cas9 proteins with high spatial specificity.

Tunable surface functionalization is another outstanding feature of gold nanocarriers that accelerates the entry of foreign genes into cells. Various biomolecules, such as proteins, DNA, peptides, and polymers, can endow gold nanomaterials with tremendous functions for surface bioengineering (Miao et al., 2018). Protamine, as a natural protein that originates from sperm, has intrinsic cell-penetrating properties and nucleus-targeting abilities and can be used for the efficient delivery of the Cas9–sgRNA plasmid. Protamine can form a compact structure with anionic DNA and then deliver the DNA to the egg nucleus (Biju et al., 2012; Priya et al., 2014). Nanocomplexes of Cas9-gRNA_EGFP_ and protamine-functionalized gold nanoclusters disrupt the EGFP gene effectively and convert approximately 30% of the EGFP-positive transformants to EGFP-negative cells (Tao et al., 2021). Meanwhile, AuNCs can be functionalized by electrostatic action to control the self-assembly process. In a highly pH-dependent manner, AuNCs assembled with Cas9 protein (SpCas9–AuNCs) can deliver SpCas9 into the cell and nucleus in physiological conditions (Ju et al., 2019). The self-assembled SpCas9–AuNC nanoparticles effectively transfect HPV18 E6 sgRNA into cervical cancer cells, knocking out the E6 oncogene at a rate of 34%. More importantly, self-assembled SpCas9–AuNCs had little effect on normal cells, showing a considerable potential for clinical application. However, concerning the application of gold nanomaterials, cytokine production, the extensive modification requirement, fewer *in vivo* experiments, and potential toxicity need to be fully considered (Dykman and Khlebtsov, 2017). Gold nanomaterials are potentially an excellent delivery system and a bright prospect for improving CRISPR systems. Additionally, they can be extensively applied to bioimaging, optical and electrochemical sensing, and medical diagnostics (Chen et al., 2016). The multifunctional integrated gold nanomaterial platform may make great contributions to biological research in the future.

#### 4.2.2 Polymers

Polymers can also be used to deliver RNP complexes to target sites with many distinct advantages, such as ease of synthesis, structural and component flexibility, functionalization, and degradability (Chen et al., 2016). Their significant flexibility is the most fascinating feature, resulting in multifunctionality by the reasonable and convenient design of the chemical structure (Hsu and Uludag, 2012; Zhang et al., 2019b). Currently, commonly used polymers to deliver drugs or RNP include polylysine, chitosan nanoparticles, poly-(β-amino ester)s, and dendrimers. The first two kinds are commonly used for drug delivery, while the latter two are mostly used for RNP delivery. Studies of drug delivery with polymers have been described in detail in other reviews (Huo et al., 2017; Hasheminejad et al., 2019). For a wide range of unmet therapeutic needs and personalized medicine, poly-(β-amino esters), as a class of amphiphilic and pH-sensitive polymers, can efficiently bind to cargo proteins to facilitate efficient intracellular RNP delivery *via* hydrogen bonding as well as hydrophobic and ionic interactions (Dwivedi et al., 2012). This characteristic allows them to be customized specifically to overcome delivery barriers in varied applications (Karlsson et al., 2020). Dendrimers are a class of synthetic polymer with a spherical and hyperbranched structure, whose surface is functionalized with a high density of phenylboronic acid moieties to ensure that RNPs are efficiently bound to the dendrimer scaffold and transmit RNP to specific cells (Dixit et al., 2014). As a novel therapeutic tool for genetic disorders, dendrimers allow the efficient delivery of RNP targeting multiple genetic loci in different cell lines, proving to be a useful material for the delivery of genome-editing tools with broad biomedical applications (Taharabaru et al., 2020). Several issues exist with RNP delivery using polymers, however, such as low efficiency, high cytotoxicity, and narrow application range, which need to be overcome in the future.

## 5 CRISPR Regulation With Nuclease-Dead Cas Proteins

Through the same mechanism mentioned above, sgRNA-directed dCas9 binds to specific DNA sequences. When dCas9 binds specifically to a genomic locus, it can sterically block or activate RNP progression to downstream genes. These two dCas9-based strategies are called CRISPR interference (CRISPRi) (Ji et al., 2020) and CRISPR activation (CRISPRa), respectively (Larson et al., 2013). Both strategies can precisely regulate the expression of the sgRNA module or dCas9 *via* an inducible expression system. As of yet, several dCas9-based CRISPRa methods have been established, including dCas9-P65AD (Gilbert et al., 2013), dCas9-VPR (Chavez et al., 2015), dCas9-p300 (Hilton et al., 2015), and dCas9-TET (Xu et al., 2018). Some CRISPRi methods have also been reported, including dCas9-KRAB (Abudayyeh et al., 2017), dCas9-LSD1/KDM1A (Gilbert et al., 2013), and dCas13-YTHDF2 (Rauch et al., 2018). Several excellent reviews concerning different dCas-based CRISPRi and CRISPRa strategies describe their mechanism and principle in detail (Kampmann, 2018; Xu et al., 2020b). Currently, they are utilized to screen cellular genomes, including for cell survival/proliferation, sensitivity to drugs or toxins, fluorescent reporters, and single-cell transcriptomes (Kampmann, 2018). They are expected to precisely regulate editing time to reduce off-target effects.

## 6 Enrichment of Mutants

Due to off-target effects, not all genetically modified cells are equipped with positive mutants *in vitro*. The selection of mutants from original gene-edited cells is still a challenge at present (Ren et al., 2015). Thus, new strategies need to be investigated for enrichment and selection (Figure 2C). The most common selection markers to enrich positive cells are fluorescent proteins, antibiotic resistance genes, cell surface antigens, and so forth. Due to the merits of visualization, time saving, and decreased labor, fluorescent proteins are widely utilized in CRISPR/Cas systems (Ren et al., 2019). For a variety of cellular and environmental contexts, the variety of fluorescent genes gives scientists immense flexibility in choosing tailored reporters, such as green fluorescent protein, red fluorescent proteins (Liu et al., 2021a), and fluorescent proteins (Cao et al., 2019). Nevertheless, isolated cells are easily damaged by the solid lasers and hydrostatic pressure of flow cytometry. Compared to fluorescent proteins, the antibiotic-based method offers an alternative strategy that does not require expensive equipment but needs more time (Moriarity et al., 2014; Liesche et al., 2016). Although numerous antibiotic resistance genes have been applied in various fields, such as hygromycin (Moriarity et al., 2014), neomycin (Gu et al., 2021), zeocin (Kobayashi et al., 2019), gentamicin (Mulsant et al., 1988) and puromycin (Pandey et al., 2021), marker-free strategies are the preferred method, ameliorating public concerns for the biosafety of antibiotic resistance genes. Another non-fluorescence activated cell sorting-based enrichment method is antigen gene H-2K^k^, which has a high enrichment efficiency with magnetic bead separation (Wei et al., 2001). However, when insertions or deletions are generated at the target sequences, these reporter systems express H-2K^k^ and hygromycin resistance protein, respectively, enabling the efficient enrichment of mutants without flow cytometry (Kim et al., 2013).

However, no matter what efficient strategies are used to select mutants, mutant enrichment alone cannot classify all stable and highly expressed mutants (Figure 2D). Thus, to select nuclear-active mutants, two surrogate reporters based on the NHEJ and single-strand annealing (SSA) have been published (Pattanayak et al., 2013). Nuclease triggers a DSB on the target sequence within the surrogate reporter construct, resulting in the formation of small random indels by the error-prone NHEJ repair pathway and leading to the correction of reporter genes with 1/3 frequency. Compared with unsorted cells, the enrichment efficiency of mutants can be increased up to 8.6- and 18-fold with the first and second generation of NHEJ-based surrogate reporters, respectively (Ramakrishna et al., 2014a). The second surrogate reporter has the capacity to identify more nuclease-positive cells *via* SSA. Due to its higher sensitivity, this reporter significantly increases the possibility of obtaining the desired genetically modified cell clones (Yasuda et al., 2016). Although DNA repair pathways are influenced by cell type and the nature of broken DNA ends, genomic modification within mutants may be independent of repair pathways in surrogate reporters (Ren et al., 2015). On the basis of transfection-positive cells, these two surrogate reporter strategies can produce highly efficient, nuclease-active cells.

## 7 Conclusion and Future Prospects

Aside from the above-mentioned approaches, other strategies can also significantly improve the editing efficiency of CRISPR/Cas systems. Firstly, owing to the fact that the nucleosome poses a strong barrier to Cas9, restoring Cas9 access to nucleosomes through the chromatin remodeling enzyme yChd1 therefore results in high efficiency editing. Nucleosome organization represents only one aspect of eukaryotic chromatin, however; thus, future research on how chromatin affects Cas9 activity needs to be done (Horlbeck et al., 2016). Secondly, cytosine base editors (CBE) and adenine base editors (ABE) have been utilized to change C/G to T/A and A/T to G/C. CBE deaminates cytosine to uracil, which is recognized by the cell replication machinery as thymine, resulting in a C/G to T/A transition. ABE-mediated DNA editing operates under a similar mechanism as that of CBE (Koblan et al., 2018; Richter et al., 2020). Despite efforts to improve DNA base editors, base editing is confined to transition mutations (incapable of transversion mutation) and is not capable of inducing indel mutations. Next, by combining reverse transcriptase with prime editors gRNA and Cas-nickase nuclease, prime editing technology can edit or “search and replace” bases in a genome (Anzalone et al., 2019). It can also be used as an alternative genome editing tool to investigate various challenges, such as editing large genes, targeting autosomal dominant diseases, and editing premature stop codons and splice-site variants (Kantor et al., 2020). When prime editors are undesirable and the base editing window is well defined, base editors are typically more efficient than prime editors. On the contrary, when prime editors are acceptable and multiple editable bases are within a defined editing window, prime editors offer unsurmountable advantages.

In the last few years, we have seen the extraordinary growth and expansion of gene editing, particularly in the field of gene therapy. Based on CRISPR technology, a series of highly efficient and targeted transcription factor components has been developed and used to construct intelligent gene circuits, making tumor gene therapy possible (Zhou et al., 2019). In cardiovascular medicine, CRISPR-based tools have multiple applications, with a primary focus on direct therapeutic interventions to treat inherited cardiac disorders (Vermersch et al., 2020). CRISPR also represents a breakthrough advance in genetically engineered immune cells (Huang et al., 2020), personalized cancer medicine (Li and Kasinski, 2020), and modification of human embryos (Tang et al., 2017). Even in the current novel coronavirus (COVID-19) outbreak, CRISPR-based technology has shown strong application value. All-in-one dual CRISPR-Cas12a is instrumental in the detection of COVID-19, offering the advantages of being instrument-free, rapid, sensitive, one-pot, and point-of-care (Ding et al., 2020). Applications in microbiology are still being newly discovered and improved, specifically in the identification and modification of industrial-related lactobacilli and streptococci as well as foodborne pathogens, including *E. coli* (Altenbuchner, 2016), *Saccharomyces cerevisiae* (Biot-Pelletier and Martin, 2016), and thermophilic fungi (Liu et al., 2017b). As a new generation of precision gene editing tools, the great success of CRISPR/Cas systems in various fields shows that these have a wide range of application and wonderful prospects.

Collectively, knowledge and technologies of genome editing are ceaselessly developing in intricately interwoven fields and are creating huge synergies. With the recent developments in CRISPR/Cas systems, they are becoming increasingly accurate, efficient, and reliable. Although massive advances have been achieved, the CRISPR/Cas systems are far from their optimal state. Among various challenges, off-target effects are still the foremost barrier in CRISPR/Cas systems. We have listed above several strategies for reducing off-target effects. Among them, special attention should be paid to optimizing time and temperature, which are often inadvertently neglected. The CRISPR/Cas systems have other limitations, including inactive mutants, variable efficiency, requirement of PAM and sgRNA, fault-prone programmed DNA repair pathways, and the lack of an efficient and safe delivery system. Apart from these, future research will involve the enhancement of Cas9 activity, application of ACR proteins, and determination of the optimal Cas9 and sgRNA ratio so as to further improve the efficiency of CRISPR systems. Simultaneously, continuous optimization of external measures, including dCas9 regulation, delivery vector development, mutant enrichment, *etc*., will help to further improve the efficiency. Although we are far from eliminating off-target effects completely, we are confident that CRISPR technology will continue to be perfected to meet the demands of different fields by adopting the aforementioned strategies.

## Glossory

CRISPR: clustered regulatory interspaced short palindromic repeats
ZFNs: zinc finger nucleases
TALENs: transcription activator-like effector nucleases crRNA
PAM: proto-spacer adjacent motifs
sgRNA: single guide RNA
DSBs: double-strand breaks
PCR: Polymerase chain reaction
Acr: anti-CRISPR
NHEJ: nonhomologous end joining
c-NHEJ: canonical NHEJ
HDR: homology-directed repair
53BP1: tumor suppressor p53-binding protein 1
CtIP: CtIP C-terminal-binding protein interacting protein
dn53BP1: dominant-negative 53BP1
ssODN: single-stranded oligodeoxynucleotides
dsDNA: double-stranded DNA
EVs: extracellular vehicles
CPPs: cell-penetrating peptides
AAV: adeno-associated virus
LV: lentivirus
AdV: adenovirus
GO: graphene oxide
TDNs: tetrahedral DNA nanostructures
CAR: chimeric-antigen receptor
LNP: lipid nanoparticles
NIR: near-infrared
HSF: heat-shock factor
AuNR: gold nanorods
AuNCs: gold nanoclusters
CRISPRi: CRISPR interference
CRISPRa: CRISPR activation
eGFP: enhanced green fluorescent proteins
RFP: red fluorescent proteins
FPs: fluorescent proteins
CBE: cytosine base editors
ABE: adenine base edito
